# Infertility as a Rare Manifestation of Pituitary Involvement in Granulomatosis With Polyangiitis: A Case Report

**DOI:** 10.7759/cureus.86780

**Published:** 2025-06-26

**Authors:** Mohammad Syedul Islam, Quazi Mamtaz Uddin Ahmed, Farzana Ahmed, Md. Ashraf Uddin, Naznin Naher

**Affiliations:** 1 Department of Internal Medicine, Bangladesh Medical University, Dhaka, BGD; 2 Department of Paediatrics, Marks Medical College and Hospital, Dhaka, BGD

**Keywords:** anca associated vasculitis, cyclophosphamide therapy, hypogonadotropic hypogonadism, infertility, pituitary dysfunction

## Abstract

Granulomatosis with polyangiitis is a rare autoimmune disease that affects small-sized blood vessels. It most commonly involves the lungs, kidneys, and the areas around the nose and throat, but can also affect other organs, including the nervous system. Enlargement of the pituitary gland may cause a mass effect, and pituitary dysfunction can occur. Among pituitary hormone abnormalities, hypogonadotropic hypogonadism is more common than other hormonal deficiencies. Pituitary involvement may arise at any stage of the disease, sometimes even as the initial presentation. We report a rare case of granulomatosis with polyangiitis with pituitary dysfunction presenting as infertility, along with pulmonary and systemic manifestations.

## Introduction

Granulomatosis with polyangiitis (GPA) is a rare systemic vasculitis characterized by necrotizing inflammation of small-sized blood vessels, with an unclear etiology. The annual incidence of GPA is estimated at 10 to 20 cases per one million individuals, with a higher prevalence reported in colder climates [1]. While GPA can affect all racial and ethnic groups, it is more frequently observed among White populations. The disease typically presents between the ages of 45 and 60 years, with a roughly equal distribution between males and females [2]. The upper and lower respiratory tracts, along with the kidneys, are the most commonly affected organs, with involvement seen in 70-100% and approximately 70% of cases, respectively [3]. The involvement of the pituitary gland is rare, occurring in approximately 1% of cases [4]. When present, it often manifests with symptoms related to mass effect, such as headaches and visual disturbances, as well as anterior and posterior pituitary hormone deficiencies [4]. Pituitary involvement may occur at any stage of the disease, including at initial presentation. Clinical and radiologic improvements have been observed following immunosuppressive therapy [5]. Among patients with pituitary dysfunction, hypogonadism and diabetes insipidus are the most frequently reported endocrine manifestations, observed in 78% and 71% of cases, respectively. Despite high systemic remission rates with maintenance immunosuppressive therapy, persistent pituitary dysfunction remains common, affecting up to 86% of patients [6].

## Case presentation

A 28-year-old woman with a prior diagnosis of GPA, initially presenting with multiple cavitary lesions and nodules in the lungs, was admitted to our department on February 11, 2025, with a two-month history of persistent headache, a 10-day history of cough, and redness in both eyes for the past two days. She had been diagnosed with GPA six months earlier, on August 15, 2024, and was treated according to the EUVAS (European Vasculitis Society) ANCA-associated vasculitis protocol. Induction therapy included pulse doses of intravenous methylprednisolone followed by six doses of cyclophosphamide (15 mg/kg). This regimen led to significant clinical improvement, evidenced by a reduction in inflammatory markers and radiologic resolution on a follow-up chest X-ray.

However, the patient missed her final two scheduled cyclophosphamide doses, with the last dose administered on November 11, 2024. She returned for follow-up in December, approximately 1.5 months later, and was initiated on maintenance therapy with oral azathioprine (100 mg daily) and prednisolone (10 mg daily). She remained in clinical remission for about one month following this regimen. At the time of initiating cyclophosphamide, she was counseled about the potential side effects of the drug, including its impact on fertility. However, due to financial constraints and the urgency of her life-threatening condition, the treatment was started.

At the time of admission, the patient presented with fever, productive cough, bilateral eye redness, and blurring of vision. She also reported generalized malaise and joint pain. Additionally, she had been experiencing a persistent, diffuse headache for the past two months, which was not associated with vomiting and had no identifiable aggravating or relieving factors. Upon further history-taking, the patient stated that she and her partner had been trying to conceive since March 2021. Despite seeking medical evaluation at multiple centers, no definitive diagnosis had been established, and she was unable to provide documentation of those consultations. She also reported irregular and delayed menstrual cycles over the past three years. A thorough review of her medication history was also conducted to identify any other potential agents that might have contributed to her reproductive issues.

**Figure 1:**
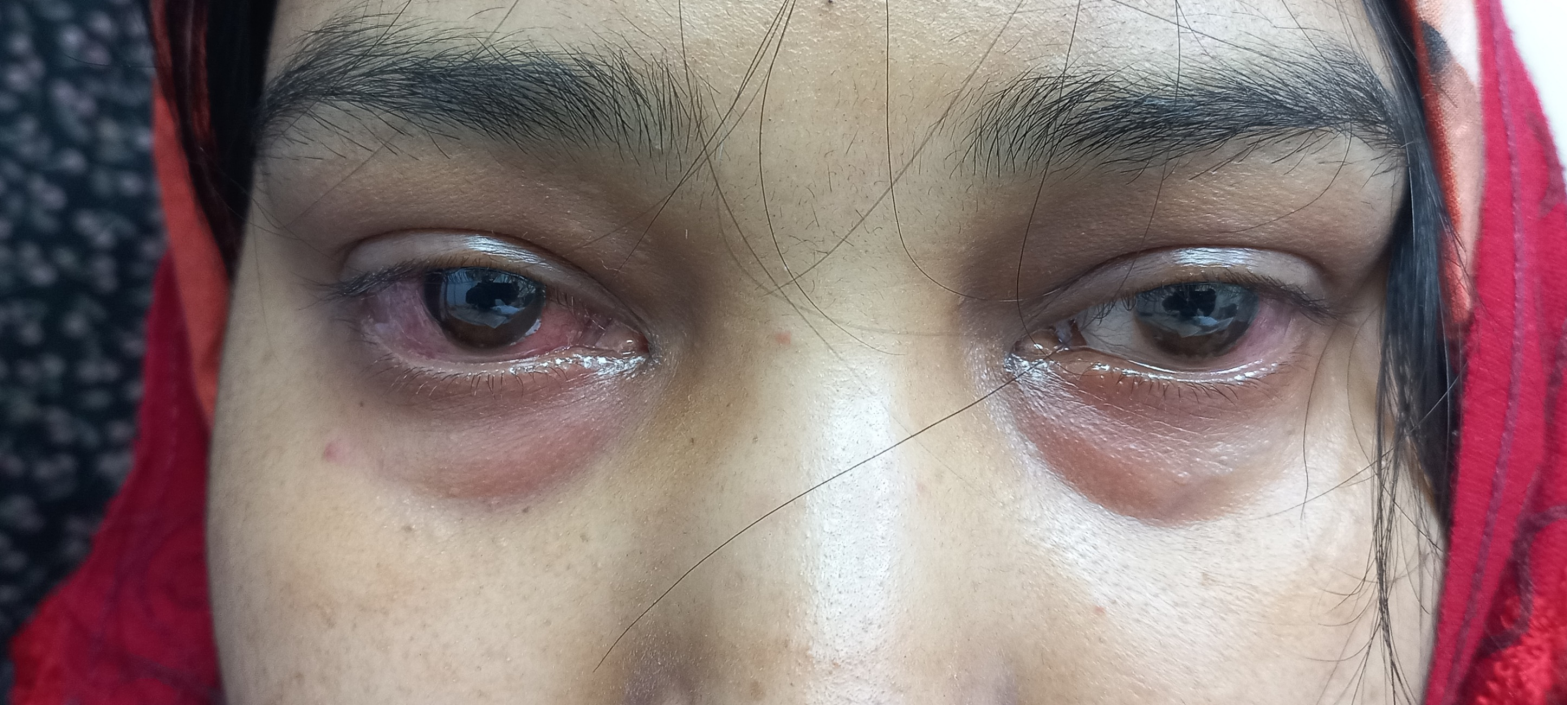
Bilateral periorbital swelling and conjunctival injection with chemosis, consistent with scleritis in a patient with GPA GPA: granulomatosis with polyangiitis

At the time of admission, she was found to be febrile (temperature: 38.3 °C) and tachypneic and had bilateral crackles on lung auscultation. Ophthalmic evaluation revealed bilateral lid swelling and chemosis, consistent with scleritis (Figure 1). There were no signs of meningeal irritation or papilledema, although some visual defects were noted.

Laboratory investigations revealed normocytic normochromic anemia (Hb 7.7 g/dL), leukocytosis (WBC 11.9 × 10³/μL with 86% neutrophils), and thrombocytosis (platelets 1100 × 10³/μL). Inflammatory markers were significantly elevated, with a CRP of 160 mg/L and an ESR of 91 mm/hour. Blood cultures were negative. Renal function was normal (serum creatinine 1.1 mg/dL), and urinalysis was unremarkable (Table 1).

**Table 1:** Laboratory findings showing markedly elevated inflammatory markers CBC: complete blood count, WBC: white blood cell count, MCV: mean corpuscular volume, MCH: mean corpuscular hemoglobin, urine RME: urine routine microscopic examination, ESR: erythrocyte sedimentation rate, CRP: C-reactive protein

Investigation	Result	Reference range
CBC		
Hb	7.7 gm/dl	11.20-15.70 gm/dl
MCV	88.9 fl	76-96 fl
MCH	28.4 pg	27-32 pg
RBC	2.71 million/dl	4.04-6.12 million/dl
WBC	11.9 × 10³/μL	4 – 11 × 10³/μL
Platelet	1100 × 10³/μL	150 – 450 × 10³/μL
ESR	91 mm in 1^st^ hour	0 – 10 mm in 1^st^ hour
CRP	160 mg/L	< 5 mg/dl
Blood C/S	Normal growth	
Urine RME	Normal study	
S. Creatinine	1.1 mg/dl	0.5-1 mg/dl

**Figure 2:**
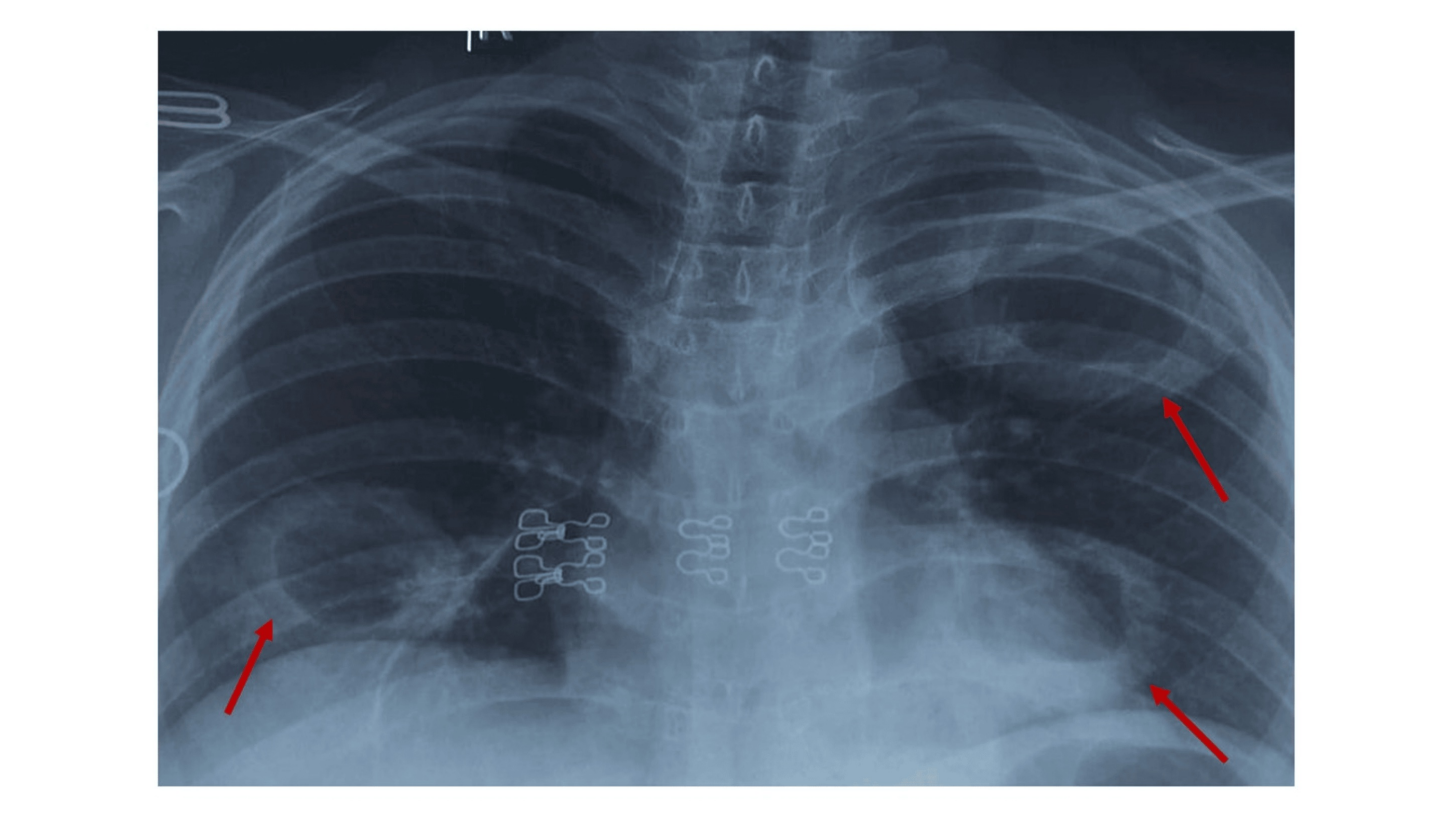
The arrows indicate multiple thick-walled cavitary lesions in the right mid-zone and the left lung field The cavities vary in size and have irregular, well-defined thick walls.

The chest X-ray showed multiple thick-walled cavitary lesions in the right mid-zone and left lung fields (Figure 2).

**Figure 3:**
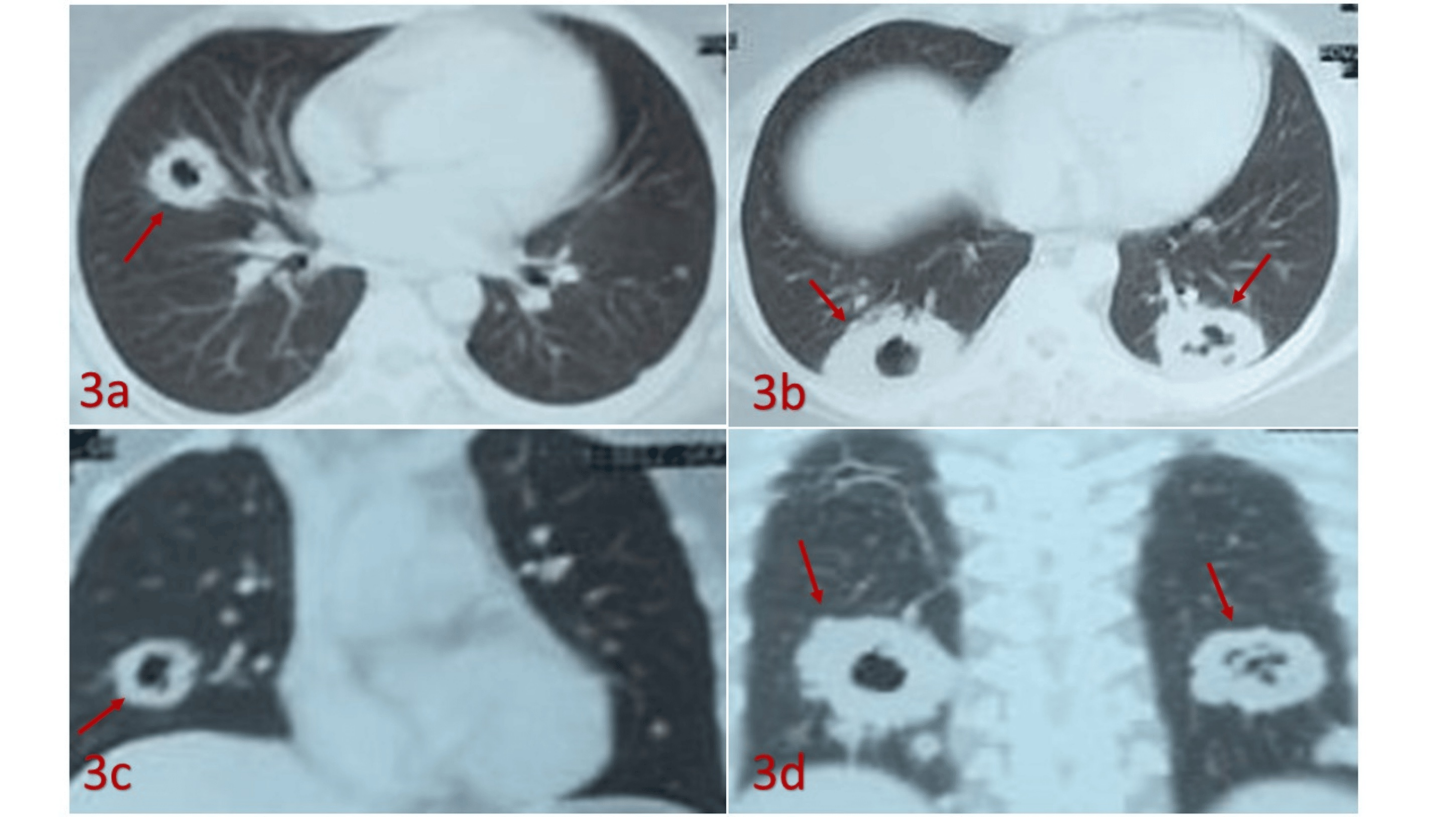
High-resolution computed tomography (HRCT) chest images (a–d) showing characteristic pulmonary findings in GPA Axial (3a, 3b) and coronal (3c, 3d) views reveal multiple thick-walled cavitary nodules (red arrows) in both lungs. These findings are suggestive of necrotizing granulomatous inflammation, typical of GPA-related pulmonary involvement. GPA: granulomatosis with polyangiitis

Contrast-enhanced CT of the chest revealed numerous cavitary lesions and nodules in multiple lung segments (Figure 3). Bronchoalveolar lavage fluid tested negative for acid-fast bacilli, GeneXpert MTB/RIF, GMS stain, PAS stain, and bacterial and fungal cultures.

Given her persistent headache and concerns about infertility, a brain MRI and hormonal profile were performed. Hormonal evaluation revealed evidence of hypopituitarism, with a normal thyroid-stimulating hormone (TSH) level of 1.20 µIU/mL (reference range: 0.4-4.0), but a slightly low free T4 of 0.78 ng/dL (0.9-2.4). Gonadotropins were low, with LH at 0.76 mIU/mL (0.5-76.3) and FSH at 3.95 mIU/mL (15-20), consistent with hypogonadotropic hypogonadism. Growth hormone was at the lower limit of normal, measuring 0.17 ng/mL (0.126-9.88). Prolactin was within normal limits at 11.22 ng/mL (2.80-29.20), making the stalk effect or prolactinoma less likely. Morning cortisol was 15.6 µg/dL; however, interpretation was limited due to chronic steroid therapy. Adrenocorticotropic hormone (ACTH) was 16.05 pg/mL (7.20-63.30). No clinical signs of antidiuretic hormone (ADH) deficiency were observed, and investigations for ADH deficiency were not performed because there were no suggestive clinical features (Table 2).

**Table 2:** Laboratory findings indicative of pituitary dysfunction with low gonadotropin levels TSH: thyroid-stimulating hormone, LH: luteinizing hormone, FSH: follicle-stimulating hormone, ACTH: adrenocorticotropic hormone

Investigation	Result	Reference range
Growth hormone	0.17 ng/ml	0.126 - 9.88 ng/ml
TSH	1.20 µIU/mL	0.4-4.0 µIU/mL
T4	0.78 ng/dL	0.9-2.4 ng/dL
Prolactin	11.22 ng/mL	2.80-29.20 ng/mL
FSH	3.95 mIU/mL	15-20 mIU/mL
LH	0.76 mIU/mL	0.5-76.3 mIU/mL
Morning cortisol	15.6 µg/dL	4.46–22.70 µg/dL
ACTH	16.05 pg/mL	7.20–63.30 pg/mL

**Figure 4:**
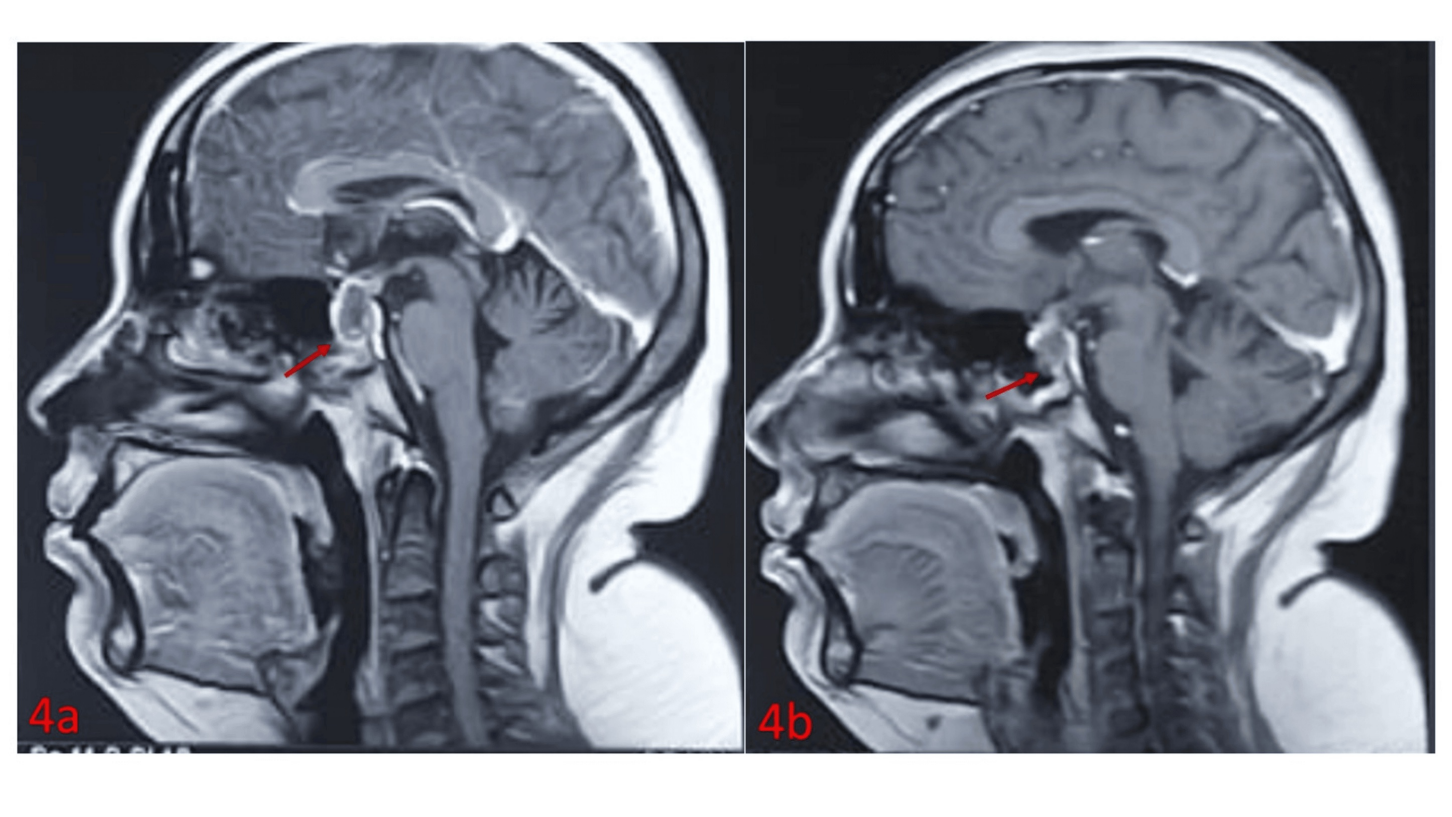
Sagittal T1-weighted contrast-enhanced MRI brain images (a–c) showing pituitary involvement in GPA The images reveal a homogeneously enhancing sellar mass with peripheral rim enhancement (red arrows), thickening of the pituitary stalk, and mild superior compression of the optic chiasm. GPA: granulomatosis with polyangiitis

Imaging revealed a homogeneously enhancing sellar mass with thickening of the pituitary stalk, causing mild compression of the optic chiasm (Figure 4).

The patient was treated with intravenous methylprednisolone (1 g daily for three days), followed by high-dose oral prednisolone at 1 mg/kg/day (total dose 70 mg/day) with a tapering regimen. Due to the urgency of her clinical condition, the previously missed fifth dose of cyclophosphamide was administered on February 16, 2025, after appropriate counseling and obtaining written informed consent. Her clinical response was prompt, with resolution of respiratory symptoms and improvement in oxygen saturation from 94% to 98% on room air. She was discharged with advice to return after 10 days for the final (sixth) dose of cyclophosphamide. At follow-up on March 1, 2025, she received the sixth dose of cyclophosphamide and was started on maintenance therapy with azathioprine (50 mg daily), along with a tapering schedule of oral prednisolone. She remained in clinical remission from that time. At her most recent follow-up, the patient was advised to undergo a repeat brain MRI and hormonal testing to assess pituitary function both biochemically and radiologically. A multidisciplinary approach was planned, involving collaboration with the endocrinology and gynecology departments for better management.

## Discussion

GPA is an ANCA-associated vasculitis that may involve the central nervous system (CNS), including the pituitary gland, meninges, and cerebral vasculature, in approximately 7-11% of cases [7]. Patients diagnosed with this manifestation have ranged in age from 28 to 67 years, with no notable difference between males and females. On average, pituitary dysfunction (PD) tends to develop approximately 10.4 months after the initial diagnosis of GPA, though this can range from 1 to 36 months [8]. Interestingly, PD was the presenting feature in about 46% of cases, particularly in younger individuals, and in approximately 2.5% of patients, it was the only clinical sign of the disease [9].

Three potential mechanisms have been suggested to account for the distinctive CNS involvement seen in granulomatosis with polyangiitis (GPA): first, the spread of inflammation from the sinonasal region into surrounding tissues; second, the direct formation of granulomatous lesions within the brain; and thirdly, a primary vasculitis targeting small-sized arteries in the CNS [10]. Hypophysitis, an inflammatory condition of the pituitary gland, presents in various histological forms, with necrotizing granulomatous hypophysitis being the most common in GPA [11]. When autoimmune vasculitis affects the pituitary gland, it may produce granulomatous inflammation that can result in a mass effect. This can manifest clinically as headaches, disturbances in vision, and deficiencies in hormones secreted by both the anterior and posterior portions of the pituitary [12]. For patients who exhibited pituitary dysfunction (PD) along with other systemic symptoms at the time of presentation, the interval between symptom onset and the diagnosis of granulomatosis with polyangiitis (GPA) averaged 1.5 months. In contrast, when PD occurred without the typical manifestations of GPA, the diagnosis of vasculitis was often delayed, sometimes taking anywhere from 3 to 36 months [13]. The use of medications such as cyclophosphamide and glucocorticosteroids may have contributed to an exaggerated frequency of hypogonadism reported in some studies. Moreover, hyperprolactinemia has been identified in about half of patients, and panhypopituitarism has been documented in approximately a quarter of cases, primarily due to compression of the pituitary stalk by an enlarged pituitary gland [14].

Currently, no standardized diagnostic criteria exist for confirming pituitary involvement in granulomatosis with polyangiitis (GPA). Diagnosis of GPA-related hypophysitis is often made when patients present with pituitary hormone deficiencies or visual disturbances, combined with positive serological markers and a known history of GPA affecting organs such as the kidneys, lungs, or sinuses. However, isolated cases of GPA confined to the pituitary gland without systemic manifestations have also been reported [15]. Gadolinium-enhanced magnetic resonance imaging (MRI) of the pituitary is considered the gold standard for radiological evaluation of pituitary involvement. According to Kapoor et al., the most frequent imaging findings in patients with pituitary GPA include a sellar mass exhibiting peripheral enhancement, central cystic areas, and compression of the pituitary stalk. Other studies have reported an enlarged pituitary gland with either uniform or heterogeneous enhancement. Additionally, thickening and increased enhancement of the infundibulum, particularly in its upper segments, are commonly observed [16]. Our patient’s score was −8, which falls within the range indicative of autoimmune hypophysitis (score ≤ 0).

Different scoring systems have been proposed in the literature to help distinguish hypophysitis from pituitary adenoma. One such system, developed by Gutenberg et al., utilizes MRI characteristics of the pituitary lesion and demonstrates a sensitivity of 92% and specificity of 99% for diagnosing autoimmune hypophysitis. This scoring method evaluates factors including lesion volume, symmetry, signal intensity, and homogeneity after gadolinium administration, the presence of the posterior pituitary bright spot, stalk thickness, mucosal swelling, patient age, and pregnancy status [17].

A pituitary biopsy is generally not necessary when there is clear clinical, serological, and systemic evidence supporting a diagnosis of granulomatosis with polyangiitis (GPA). However, a biopsy may be warranted to confirm the diagnosis if the clinical presentation is atypical or if other causes of a pituitary mass need to be excluded. Histological examination typically reveals granulomatous inflammation and inflammatory cell infiltrates, though these findings are often nonspecific in GPA-related pituitary tissue. Given its invasive nature, pituitary biopsy should be reserved for patients lacking classic GPA manifestations or surrogate markers, such as involvement of the upper or lower respiratory tract or kidneys, or for those who do not respond to conventional therapies, especially when ANCA testing is negative [18].

Because GPA-related hypophysitis is uncommon, there is a lack of studies establishing the best immunosuppressive treatment strategies, and major international guidelines (such as those from ACR, KDIGO, and EULAR) do not offer specific recommendations. Nonetheless, case reports suggest that pituitary involvement in GPA often responds well to immunosuppressive therapy. In one series of eight patients, all achieved remission following induction with corticosteroids combined with either cyclophosphamide or rituximab [6]. For maintaining remission, evidence supports the superior efficacy of scheduled rituximab infusions over conventional immunosuppressants, particularly in patients with relapsing disease. Rituximab can be given at fixed intervals of four to six months or adjusted based on B-cell or ANCA monitoring, and this approach is recommended where rituximab is accessible [19].

Despite treatment, necrotizing granulomatous inflammation leads to permanent pituitary damage in 62.5% to 86% of patients, with minimal improvement in pituitary function [6]. While systemic symptoms and pituitary imaging may improve with therapy, restoration of hormonal function is infrequent [5].

## Conclusions

This case highlights the importance of considering pituitary involvement in GPA patients presenting with endocrine symptoms such as infertility. Early diagnosis and immunosuppressive treatment may improve systemic disease and imaging findings, though pituitary dysfunction often persists. Multidisciplinary follow-up, including endocrinology and reproductive specialists, is essential for optimizing long-term outcomes and quality of life in these patients.
